# High expression of PSMC2 promotes gallbladder cancer through regulation of GNG4 and predicts poor prognosis

**DOI:** 10.1038/s41389-021-00330-1

**Published:** 2021-05-20

**Authors:** Dawei Zhu, Xing Gu, Zhengyu Lin, Dandan Yu, Jing Wang

**Affiliations:** 1 Department of Gynaecology and Obstetrics, Daping Hospital, Army Medical University, Chongqing, China; 2 Cancer Center, Union Hospital, Tongji Medical College, Huazhong University of Science and Technology, Wuhan, China

**Keywords:** Biliary tract cancer, Cell biology

## Abstract

Gallbladder cancer (GBC) is a common malignant tumor of the biliary tract, which accounts for 80–95% of biliary tumors worldwide, and is the leading cause of biliary malignant tumor-related death. This study identified PSMC2 as a potential regulator in the development of GBC. We showed that PSMC2 expression in GBC tissues is significantly higher than that in normal tissues, while high PSMC2 expression was correlated with more advanced tumor grade and poorer prognosis. The knockdown of PSMC2 in GBC cells induced significant inhibition of cell proliferation, colony formation and cell motility, while the promotion of cell apoptosis. The construction and observation of the mice xenograft model also confirmed the inhibitory effects of PSMC2 knockdown on GBC development. Moreover, our mechanistic study recognized GNG4 as a potential downstream target of PSMC2, knockdown of which could aggravate the tumor suppression induced by PSMC2 knockdown in vitro and in vivo. In conclusion, for the first time, PSMC2 was revealed as a tumor promotor in the development of GBC, which could regulate cell phenotypes of GBC cells through the interaction with GNG4, and maybe a promising therapeutic target in GBC treatment.

## Introduction

Gallbladder cancer (GBC) is a malignant tumor of the biliary tract, the common pathological type of which in the clinic is adenocarcinoma^1^. GBC accounts for 80–95% of biliary tumors worldwide, and is the leading cause of biliary malignant tumor-related death. Currently, radical resection is still the only treatment that can permanently cure GBC or significantly improve the long-term prognosis of GBC patients^2,3^. However, due to the lack of specific diagnostic biomarkers, most patients have progressed to the advanced stage when diagnosed with GBC, thus losing the opportunity for surgical treatment. In addition, because that the gallbladder wall is relatively thin, lacks serosal layer barrier, and is adjacent to the liver, it is prone to form invasion and metastasis in the liver or surrounding organs in the early stage of the disease. Therefore, despite the recent advances in the treatment of GBC, the final outcome of most patients is recurrence or distant metastasis, as well as a low 5-year survival rate and poor prognosis^4,5^. In the past decades, with the deepening of molecular tumor biology research, the concept of molecular targeted therapy was proposed and it has been successfully applied to the treatment of various tumors, and has made amazing achievements^6,7^. However, due to the lack of understanding of the molecular mechanism in the occurrence and development of GBC, the study of molecularly targeted drugs for GBC is still in the clinical trial phase^8,9^. Therefore, the exploration of key regulator in GBC which could be used as specific therapeutic target for targeted therapy is of emergency for GBC patients.

The key biological behaviors such as cell cycle progression^10^, apoptosis^11^, metabolic regulation^12^, and signal transduction^13^ have been determined to require the involvement of 26 S proteasome. Furthermore, the substrate of 26 S proteasome has attracted extensive attention. As a member of the 19 S regulatory subunit of 26 S proteasome, PSMC2 (proteasome 26 S subunit ATPase 2) is located in 7q22.1–q22.3 of the genome and is responsible for the expansion and translocation of the substrate into 20 S proteasome^14^. In addition, since the assembly of the 26 S proteasome is limited by the 19 S regulatory subunit level^15^, the expression of PSMC2 may be a necessary condition for the assembly of 19 S and 26 S proteasomes. Nijhawan et al. listed PSMC2 as the highest-ranked gene of CYCLOPS (Copy-number alterations Yielding Cancer Liabilities Owing to Partial loss), indicating a significant correlation with cancer cell activity. In addition, PSMC2 genome deletions have been found in more than 3000 types of tumors, making the remaining PSMC2 particularly important for cancer cells^15^. Not surprisingly, the role of PSMC2 in a number of human cancers has also been verified in the previous studies^16^. For example, the expression of PSMC2 is upregulated in the tumors of p21-HBx transgenic mice, whose downregulation restrains the proliferation of ovarian cancer cells^17^. Moreover, the role of PSMC2 in the progression of osteosarcoma cells is also considered to be similar to tumor-promoting factor^18,19^. Although PSMC2 has been recognized to be closely related to human cancer, its relationship with GBC has not been confirmed.

To the best of our knowledge, this study is the first to elucidate the role and mechanism of PSMC2 in GBC. Firstly, the differential expression of PSMC2 in GBC and normal tissues was determined by immunohistochemical staining of clinical specimens. Subsequently, the statistical analysis of the correlation between the expression of PSMC2 and the tumor characteristics, survival of patients with GBC showed the role of PSMC2 in the development of GBC. In addition, GBC cells with PSMC2 knockdown were examined for cell proliferation, colony formation, and apoptosis. Moreover, the potential mechanism of PSMC2 promoting GBC was further explored and verified in this study.

## Materials and methods

### Cell culture and clinical tissue microarray

Human gallbladder carcinoma cell lines, including NOZ, GBC-SD, and SGC-996 cells, were purchased from American Type Culture Collection. NOZ was cultured in CM9-1 which containing 90% DMEM-H/F12 and 10% fetal bovine serum (FBS, Vian-Saga Biotechnology). GBC-SD and SGC-996 cells were maintained in 90% RPMI-1640 (Corning Company) with 10% FBS. The tissue microarray of gallbladder carcinoma tissues and the normal tissues were purchased from Shanghai Outdo Biotech (#HGla-Ade100PG, including 78 gallbladder carcinoma tissues and 18 normal tissues; #HGal-Ade100PG-01, including 78 gallbladder carcinoma tissues and 16 normal tissues). Informed consent forms were obtained from all donors and this study was approved by the ethics committee of Huazhong University of Science and Technology.

### Immunohistochemical staining

The paraffin clinical tissue microarray was baked in an oven at 65 °C for 30 min, dewaxed in xylene, washed in descending series of alcohol, and then repaired with EDTA (Beyotime Biotechnology) in boiling water for 30 min. The sections were blocked with 3% H_2_O_2_ and related 5% serum, then incubated with appropriate primary and second antibodies. DAB dyeing solution was performed for 5 min in the dark and the staining was terminated, then counterstained by hematoxylin (Baso Diagnostics). The sections were dehydrated by 100% ethanol and sealed with neutral resin (China National Pharmaceutical Group). Finally, photographs were taken under a microscope and graded according to the staining results. All slides were examined randomly by two independent pathologists. Staining percentage scores were classified as: 1–4 (1–24%; 25–49%; 50–74%, and 75–100%). Staining intensity was scored 0–3 (Signalless color, light yellow, brown and dark brown). IHC scores were determined by staining percentage scores and staining intensity scores. Antibodies used in this study are listed in Table S1.

### Lentivirus RNAi construction and infection

The RNA interference sequences of the target gene PSMC2 and GNG4 were designed in Shanghai Bioscienceres, Co., Ltd. shPSMC2 and shGNG4 sequences were combined with vector plasmids, and then transformed into competent cells. The endotoxin-free plasmid kit was used to extract plasmids, and the qualified plasmids were transfected into 293 T cells to construct lentivirus vectors containing target sequences. All the sequences used in this study are listed in Table S2.

GBA-SD and SGC-996 cells were seeded into a 6-well plate (3 × 10^5^ cells/well), and 1 × 10^8^ TU/mL lentivirus vectors were added with ENI.S and Polybrene at a MOL of 40 for infection. After 72 h, the lentivirus infection efficiency was observed under fluorescence microscope.

### Real-time quantitative PCR (qRT-PCR)

When the cell density reached 80%, GBA-SD and SGC-996 cells were collected and the total RNA was extracted by Trizol reagent (Invitrogen). The concentration and quality of the extracted total RNA were determined by Nanodrop 2000/2000C spectrophotometer (Thermo Fisher Scientific). The cDNA was obtained by reverse transcription using the Promega M-MLV Kit (Promega Corporation) according to the manufacturer’s instruction. The forward and reverse primers used in PCR are detailed in Table S3. Two-step real-time PCR was used to determine the RNA. Melting curve Analysis was performed and the formula 2^-∆∆Ct^ was used for relative quantitative analysis, using GAPDH as the reference control.

### Western blotting and co-immunoprecipitation

The protein of GBA-SD and SGC-996 cells infected with shCtrl or shPSMC2 were collected using lysis buffer and the concentration was determined by BCA Protein Assay Kit (HyClone-Pierce). 10% SDS-PAGE was used for protein separation. Then the proteins were transferred to the PVDF membranes (Millipro Life Science). The membranes were incubated with primary antibodies after blocking with TBST solution containing 5% skimmed milk. Then the membranes were incubated with second antibody. Solution A and B of ECL-plusTM Western blot system Kit (Amersham Biosciences) were used for color development and the density of the proteins band was analyzed by ImageJ software. For co-IP, GBC-SD cells were overexpressed with Flag-labelled PSMC2 or HA-labelled GNG4. Immunocomplexes were obtained through antibodies of Flag or HA, and were analyzed with corresponding antibodies. The antibodies used are shown in Table S1.

### MTT assay

Lentivirus transfected GBA-SD and SGC-996 cells in logarithmic growth phase were digested, suspended and added into 96-well plate (2,000 cells/well) for culturing. A total of 20 μL 5 mg/mL MTT solution (Genview) was added into each well for coloring at day of 1, 2, 3, 4, and 5. After 4 h culture termination, the culture medium was discarded and 100 μL DMSO solution (Shanghai Shiyi Chemical Reagent) was added. OD value was detected at 490 nm and 570 nm wavelength with microplate reader (Tecan Infinite).

### Colony formation assay

Lentivirus transfected GBA-SD and SGC-996 cells in the logarithmic growth phase were inoculated into a 6-well plate (1000 cells/well) and cultured with the culture medium changed every 3 days. After photographed by fluorescence microscopy, the cell clones were fixed by 4% paraformaldehyde and stained with 500 μL GIEMSA (DingGuo Biotechnology). Clones were observed and number of colonies was counted (a cluster including more than 50 cells as a colony) under the microscope.

### Flow cytometry

Lentivirus transfected GBA-SD and SGC-996 cells were seeded into 6-well plates until cell density reached 85%. Cells were harvested and washed with 4 °C ice-cold PBS. After centrifugation (1,200 × g), cells were resuspended with 100 μL binding buffer and stained by 10 μL Annexin V-APC (eBioscience) at room temperature for 15 min without light. Then 400 μL binding buffer was added into the suspension, flow cytometry (Millipore) was used for detection of cell apoptosis.

### Celigo cell counting assay

After 72 h of the lentivirus infection, GBC-SD cells were collected and seeded into a 96-well plate (2,000 cells/well) and further cultured in RPMI-1640 medium containing 10% FBS at 37 °C with 5% CO_2_ for 120 h. Cell counting was accomplished by Celigo image cytometer (Nexcelom Bioscience) at day 1, 2, 3, 4, 5 and the cell proliferation was measured.

### Transwell assay

The migration ability of cells was analyzed by Transwell assay using Corning Transwell Kit (Corning). Briefly, exponentially growing lentivirus infected GBC-SD cells were collected, trypsinized, resuspended, and incubated in a 24-well plate with 5 × 10^4^ cells/well in the upper chamber with 100 μL medium without FBS. The lower chamber was felled with 600 μL medium supplemented with 30% FBS. After incubated for 24 h, non-metastatic cells were removed with a cotton swab and metastatic cells were fixed by 4% formaldehyde and stained by Giemsa. Pictures were collected and the migration ability of cells was analyzed.

### Wound-healing assay

Lentivirus infected GBC-SD cells were seeded onto a 96-well dish and cultured until the cell confluence reached over 90%. Scratches across the cell layers were made by a 96-wounding replicator (VP scientific). Floating cells were gently washed 2–3 times with a serum-free medium. Scratched cell layers were further cultured for 24 h in 0.5% FBS containing RPMI-1640 medium. Photographs were collected under a fluorescence microscope at 8 h and 24 h and the cell migration rate of each group were calculated.

### Human apoptosis antibody array analysis

Human apoptosis antibody array (Abcam) was used to detect the effect of PSMC2 knockdown on the apoptosis pathway-related protein expression. GBC-SD cells infected with shPSMC2 were cleaved with lysis buffer. The membranes were incubated with 1.2 mL cell lysate overnight at 4 °C, and then the liquid was discarded. Membranes were washed with Wash Buffer and incubated with Bolin-conjugated Anti-Cytokins overnight at 4 °C. The equal volume detection buffer C and D were mixed evenly and added to the membranes for incubation for 2 min at room temperature. The signals were detected by chemiluminescence imaging system.

### RNA screening analysis

The detection of gene expression profile in GBC-SD cells transfected with shPSMC2 or shCtrl by RNA screening analysis was completed in Shanghai Bioscienceres, Co., Ltd. Total RNA was extracted by the RNeasy kit (Sigma). Concentration and quality of total RNA were determined by Nanodrop 2000 (Thremo Fisher Scientific). RIN value was evaluated with Agilent 2100 and Agilent RNA 6000 Nano Kit (Agilent). RNA sequencing was performed with Affymetrix human GeneChip PrimeView according to the manufacturer’s instruction and the outcomes were scanned by Affymetrix Scanner 3000 (Affymetrix). Raw data statistical significance assessment was accomplished using a Welch t-test with Benjamini-Hochberg FDR ( | Fold Change | ≥ 2.0 and FDR < 0.05 as significant). Significant difference analysis and functional analysis based on Ingenuity Pathway Analysis (IPA) (Qiagen) was executed, and |Z - score | > 2 is considered meaningful.

### Mice tumor formation model

The animal experiments were approved by Huazhong University of Science and Technology. The lentivirus transfected (shCtrl and shPSMC2) GBC-SD cells in logarithmic growth phase were digested with trypsin and suspended into cell suspension. 0.2 mL cell suspension with 5 × 10^6^ cells were subcutaneously injected into the 4-week old BALB-c nude mice which were purchased from Shanghai Lingchang Biotechnology. These mice were randomly divided into shCtrl and shPSMC2 groups with 10 in each group. The tumor volume was measured 2 times per week with Vernier caliper. 0.7% sodium pentobarbital (10 ml/g) was injected intraperitoneally for in vivo fluorescence imaging. The anesthetized mice were placed under the Perkin Elmer IVIS Spectrum and images were collected. Finally, anesthetized mice were sacrificed by cervical dislocation and tumors were removed.

### Ki-67 staining assay

For Ki-67 immunostaining, tumor tissues were fixed and embedded with formalin and paraffin. A total of 5 μm slides were cut and immersed in xylene and 100% ethanol for deparaffinization and rehydration, then all slides were blocked with PBS-H_2_O_2_. All slides were incubated with Ki-67 primary antibody and further incubated with the secondary antibody. All slides were stained by Hematoxylin and Eosin (H&E staining, Baso). Stained slides were examined with a microscopic.

### Statistical analysis

All the above cell experiments were in triplicate. All data were analyzed by GraphPad Prism 6 (San Diego) and SPSS 17.0 (IBM) and presented in the form of mean ± SD. Student *t*-test and One-way ANOVA were used for statistical analysis, and *P* < 0.05 was considered to be significant. Rank Sum test analysis, Mann–Whitney U analysis, and Spearman Rank correlation analysis were used.

## Results

### PSMC2 was upregulated in GBC tissues and highly expressed in GBC cells

**Fig. 1:**
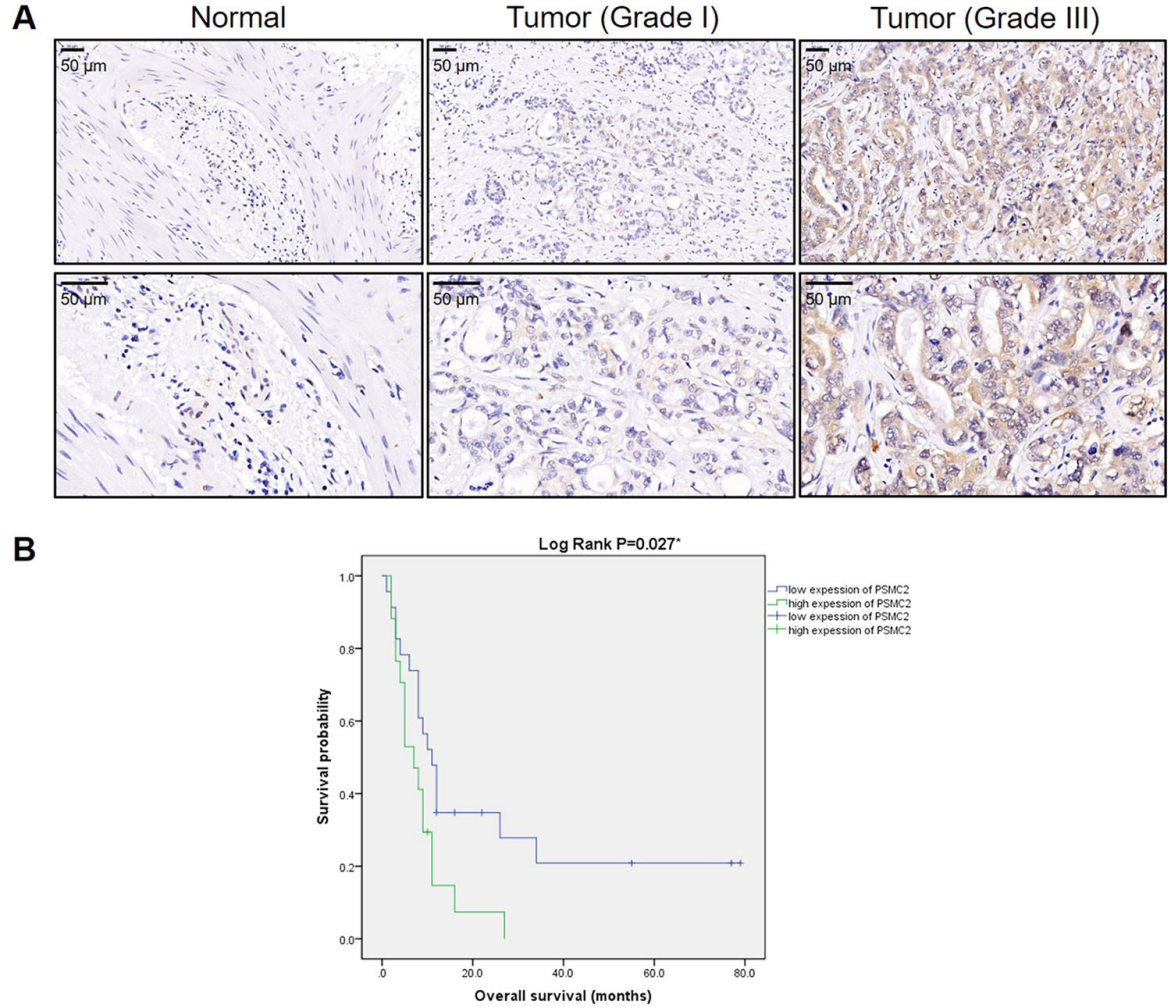
PSMC2 was upregulated in GBC tissues and predicted poor prognosis. **A** The expression level of PSMC2 was detected by IHC analysis in GBC tissues and normal tissues. **B** The relationship between PSMC2 expression and prognosis of GBC patients was statistically analyzed by Kaplan–Meier survival analysis.

To better explore the role of PSMC2 in GBC, IHC analysis was adopted to visualize the expression levels of PSMC2 in GBC tissues in comparison with regular tissues, revealing the upregulated appearance of PSMC2 in GBC (Fig. 1A). As shown by the statistical analysis of expression data collected from 78 GBC tissues and 18 normal tissues, there was a generally higher expression of PSMC2 in GBC (*P* < 0.001, Table 1). Correlation analysis between PSMC2 expression and clinical characteristics in patients with GBC revealed that PSMC2 expression was notably upregulated in patients suffering from a more advanced tumor grade (*P* < 0.05, Table 2), which can be observed in Fig. 1A and was further verified through performing a Spearman rank correlation analysis (Table S4). Together, the higher expression of PSMC2 in more serious GBC suggested its potential role in the promotion of GBC. Moreover, on the ground of Kaplan–Meier survival analysis (Log rank *P* < 0.05, Fig. 1B), we conclude that high expression of PSMC2 in tumors predicts a relatively shorter survival period.

**Table 1:** Expression patterns of PSMC2 in gallbladder cancer tissues and normal tissues revealed in immunohistochemistry analysis.

PSMC2 expression	Tumor tissue		Normal tissue	
	Cases	Percentage	Cases	Percentage
Low	41	52.6%	18	100%
High	37	47.4%	0	–

*P* < 0.001

**Table 2:** Relationship between PSMC2 expression and tumor characteristics in patients with gallbladder cancer.

Features	No. of patients	PSMC2 expression		*P* value
		low	high	
All patients	78	41	37	
Age (years)				0.324
<65	36	17 (41.4%)	19 (51.3%)	
≥65	41	24 (58.5%)	17 (45.9%)	
Gender				0.965
Male	23	12 (29.2%)	11 (29.7%)	
Female	55	29 (70.8%)	26 (70.3%)	
Grade				< 0.001
I	3	3 (7.3%)	0 (0%)	
II	30	26 (63.4%)	4 (10.8%)	
III	45	12 (29.3%)	33 (89.2%)	
T Infiltrate				0.088
T1	5	4 (9.8%)	1 (2.7%)	
T2	23	14 (34.1%)	9 (24.3%)	
T3	30	15 (36.6%)	15 (40.5%)	
T4	2	0 (0%)	2 (5.4%)	
lymphatic metastasis (N)				0.675
N0	45	24 (58.5%)	21 (56.8%)	
N1	7	5 (12.2%)	2 (5.4%)	
N2	6	3 (7.3%)	3 (8.1%)	
AJCC stage				0.345
1	4	3 (7.3%)	1 (2.7%)	
2	13	7 (17.1%)	6 (16.2%)	
3	18	11 (26.8%)	7 (18.9%)	
4	16	7 (17.1%)	9 (24.3%)	
Tumor size				0.912
<4.5 cm	38	20 (48.8%)	18 (48.6%)	
≥4.5 cm	37	19 (46.3%)	18 (48.6%)	
Lymph node positive				0.603
=0	45	24 (58.5%)	21 (56.7%)	
å 0	13	8 (19.5%)	5 (13.5%)	
Metastasize (M)				0.863
M0	68	36 (87.8%)	32 (86.5%)	
M1	10	5 (12.2%)	5 (13.5%)	

### Knockdown of PSMC2 inhibited GBC development in vitro

**Fig. 2:**
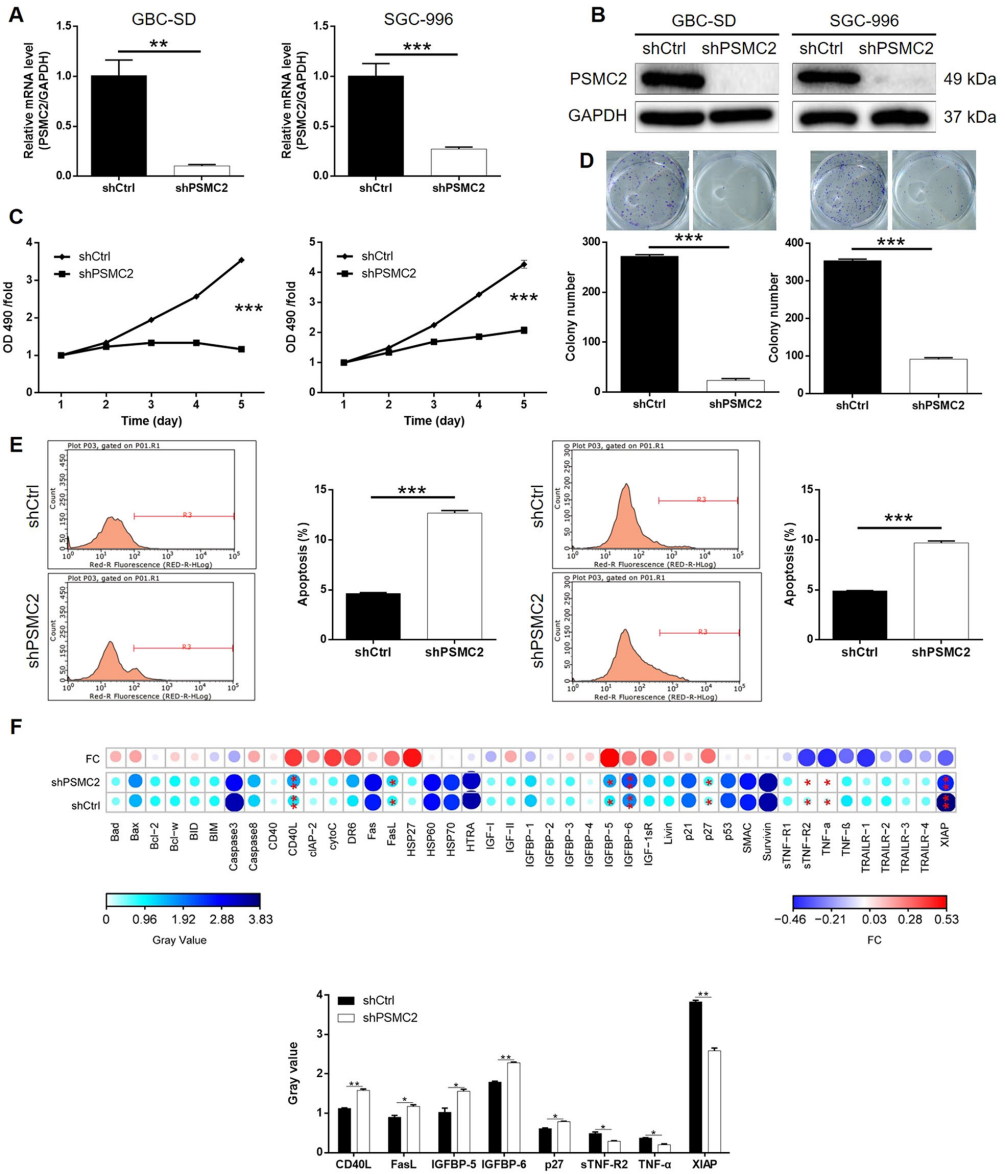
PSMC2 knockdown inhibited GBC development in vitro. **A**, **B** Cell models with or without PSMC2 knockdown were constructed by transfecting shPSMC2 or shCtrl. The knockdown efficiency of PSMC2 in GBC-SD and SGC-996 cells was assessed by qPCR (**A**) and western blotting (**B**). **C** MTT assay was employed to show the effects of PSMC2 on cell proliferation of GBC-SD and SGC-996 cells. **D** Plate clone formation assay was applied to investigate the effects of PSMC2 knockdown on colony formation ability of GBC-SD and SGC-996 cells. **E** Flow cytometry was performed to detect cell apoptosis of GBC-SD and SGC-996 cells with or without PSMC2 knockdown. **F** Human apoptosis antibody array was utilized to analyze the regulatory ability of PSMC2 on the expression of apoptosis-related proteins in GBC-SD cells. Data were shown as mean ± SD. *P < 0.05, **P < 0.01, ***P < 0.001.

A PSMC2 deficiency cell model was fabricated based on human GBC cell lines GBC-SD and SGC-996 through the transfection of lentivirus created for silencing PSMC2 to expound its detailed function within GBC. The fluorescence signal observed in >80% cells demonstrates successful transfection (Fig. S1), combined with downregulation of PSMC2 mRNA and protein levels shown by qPCR (Fig. 2A) and western blotting (Fig. 2B), confirmed the successful knockdown of PSMC2 in both GBC-SD and SGC-996 cells. MTT assay outcomes showed that cells with PSMC2 knockdown (shPSMC2) grew at a much slower rate than those without PSMC2 knockdown (shCtrl) (*P* < 0.001, Fig. 2C). Meanwhile, the ability of GBC cells to form colonies was also seriously blocked by PSMC2 knockdown (*P* < 0.001, Fig. 2D). As a key element in cell proliferation, cell apoptosis of GBC cells with or without PSMC2 knockdown was evaluated using flow cytometry. In line with assumptions, cells with PSMC2 deficiency revealed greater apoptotic cell population in comparison with shCtrl group (*P* < 0.001, Fig. 2E). Otherwise, for the sake of expounding the mechanism of PSMC2 to regulate cell apoptosis, an antibody array was performed to discriminate the influenced apoptosis-related proteins by PSMC2. It was demonstrated that PSMC2 reduction induced the expression upregulation of CD40L, FasL, IGFBP-5, IGFBP-6, p27, and downregulation of sTNF-R2, TNF-α, and XIAP (Figs. S2 and 2F). In sum, we assume that PSMC2 may play a vital role in the development of GBC through regulating cell apoptosis, colony formation, and cell apoptosis.

### GBC regulated by PSMC2 by targeting GNG4

**Fig. 3:**
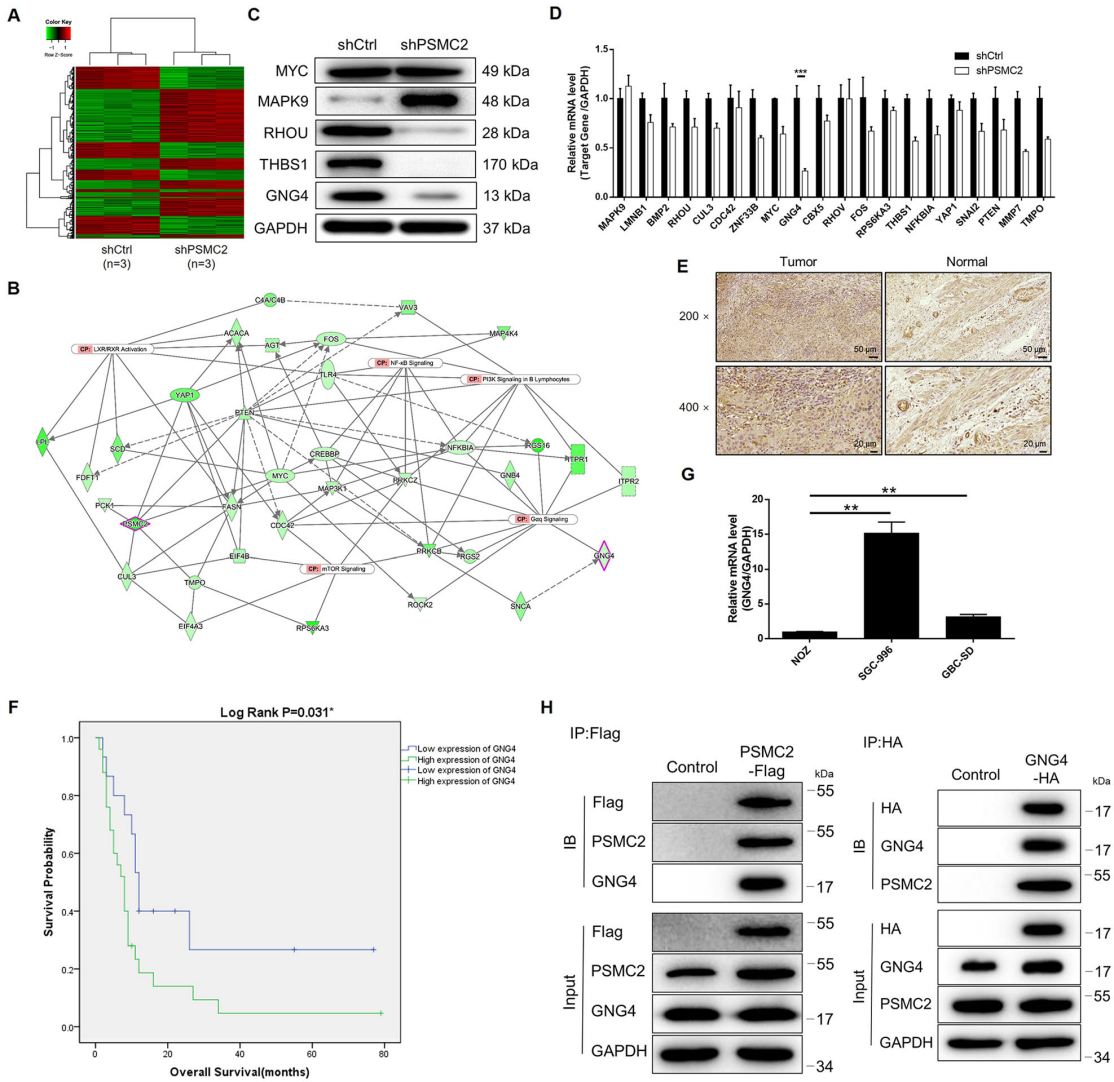
The exploration and verification of downstream underlying PSMC2 induced regulation of GBC. **A** Primeview human gene expression array was performed to identify the differentially expressed genes (DEGs) between shPSMC2 and shCtrl groups of GBC-SD cells. **B** A PSMC2-induced interaction network was established based on IPA analysis. qPCR (**C**) and western blotting (**D**) were used to detect the expression of several selected DEGs in GBC-SD cells with or without PSMC2 knockdown. **E** The expression of GNG4 in GBC tissues and normal tissues was evaluated by IHC analysis. **F** Kaplan–Meier survival analysis showed that high expression of GNG4 predicts poor prognosis of GBC patients. **G** The mRNA expression of GNG4 in GBC cell lines was detected by qPCR. The representative images were selected from at least 3 independent experiments. **H** Co-IP assay was performed to show the direct interaction between PSMC2 and GNG4. Data were shown as mean ± SD. **P < 0.01, ***P < 0.001.

Given the clear regulatory role of PSMC2 in GBC, we remained unclear over the nature of the underlying mechanism. Therefore, a 3 v 3 RNA-seq was performed to identify differentially expressed genes (DEGs) amongst cells in shPSMC2 group and shCtrl group of GBC-SD cells. Based on the threshold of simultaneous |Fold Change |≥1.3 and FDR < 0.05 (the *P* value after Benjamini–Hochberg analysis), 1177 DEGs were found to be upregulated in shPSMC2 cells associated with shCtrl cells and 851 DEGs were downregulated (Fig. S3A, Fig. 3A). The enrichment of all the 2028 DEGs in canonical signaling pathway or IPA disease and function was assessed through an IPA analysis (Fig. S3B, C). Several DEGs with the highest expression fold change were subjected to verification by qPCR and western blotting in GBC-SD cells as revealed by all the bioinformatics and the IPA-based analysis of PSMC2-associated interaction network (Fig. 3B–D). Also, gene knockdown cell models were constructed correspondingly and used for evaluating the regulatory effects of these candidates on GBC cell proliferation (Fig. S3D). Among them, GNG4, whose expression was significantly downregulated in GBC-SD cells with PSMC2 knockdown, was hypothetically the most promising contender as the target of PSMC2 because of its strongest ability to regulate GBC cell proliferation. Indeed, the expression of GNG4 showed a similar pattern with PSMC2 in GBC tissues: higher expression in GBC tissues than normal tissues (Fig. 3E and Table S5). Moreover, statistical analysis showed that GNG4 expression was also significantly associated with tumor grade and patient’s prognosis, which was similar to PSMC2 (Table S6 and Fig. 3F). The expression of GNG4 in GBC cells was also revealed by qPCR (Fig. 3G). Through the co-IP assay detecting the IP complex with anti-Flag antibody or anti-HA antibody, the interaction between PSMC2 and GNG4 was revealed (Fig. 3H). In short, GNG4 was recognized as a possible target of PSMC2 during regulating GBC, which would be further verified by in vitro investigations.

### PSMC2 Knockdown aggravated the inhibition of GBC by GNG4 knockdown

**Fig. 4:**
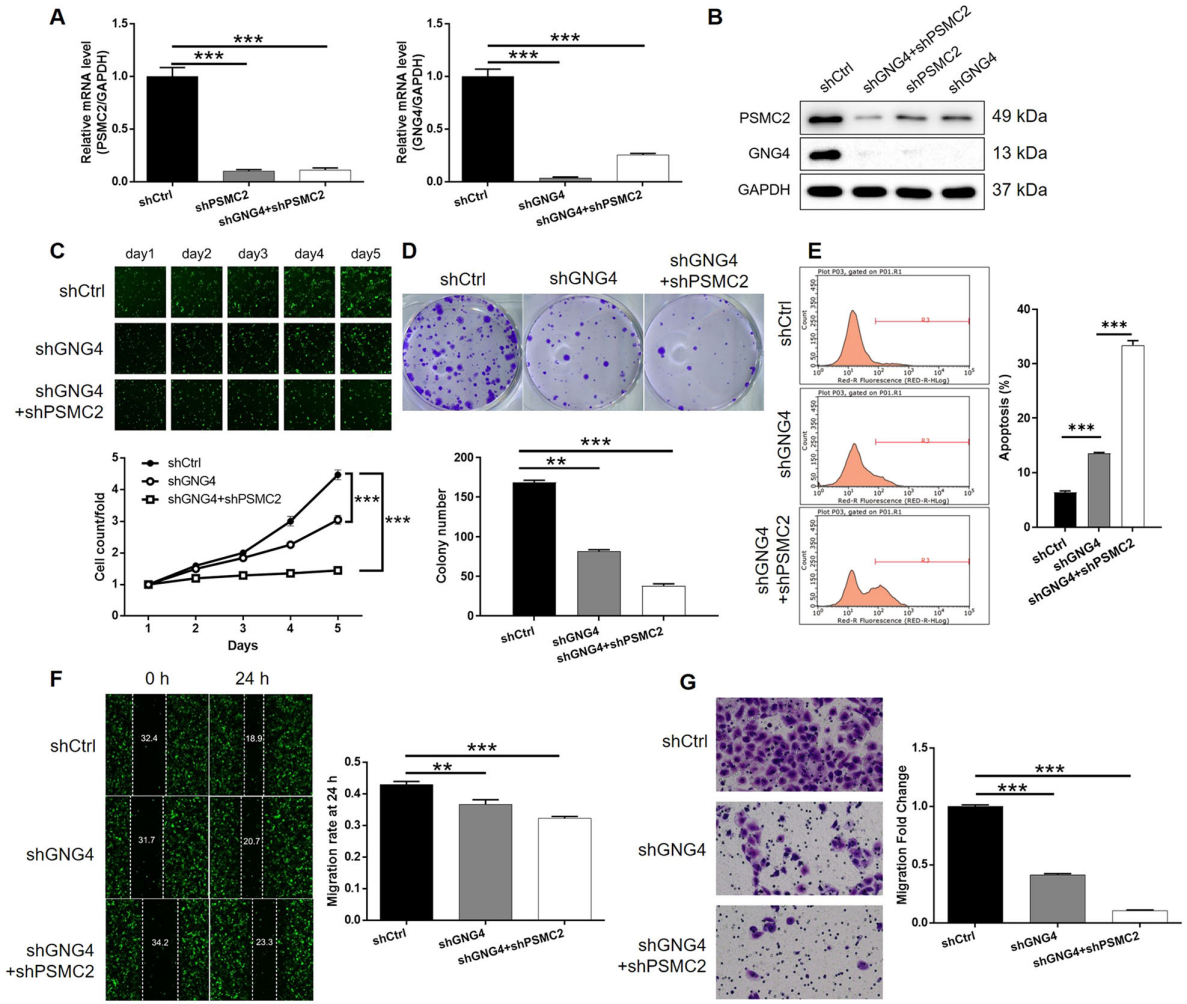
Knockdown of PSMC2 deepens the effects on GBC-SD cells by GNG4 knockdown. **A**, **B** The expression of PSMC2 and GNG4 in GBC-SD cells transfected with shCtrl, shGNG4, and simultaneous shPSMC2 and shGNG4 were detected by qPCR (**A**) and western blotting (**B**). Cell models were subjected to the detection of cell proliferation by Celigo cell counting assay (**C**), colony formation (**D**), cell apoptosis (**E**), cell migration by wound-healing assay (**F**) and cell migration by Transwell assay (**G**). The representative images were selected from at least 3 independent experiments. Data were shown as mean ± SD. **P < 0.01, ***P < 0.001.

Throughout subsequent investigations, GBC-SD cell model transfected with mere shGNG4 or both shPSMC2 and shGNG4 were established to reveal their combined action within GBC. Firstly, the transfection efficiency was evaluated by the method described above and the most effective shRNA for silencing GNG4 was screened by qPCR (Fig. S4). As shown in Fig. 4A, B, the mutual regulation between PSMC2 and GNG4 was derived: PSMC2 knockdown downregulated GNG4 while GNG4 deficiency downregulated PSMC2 (Fig. 4A, B). Additionally, the subsequent detection showed strong inhibitory effects of GNG4 knockdown on cell proliferation and colony formation as well as simulative effects on cell apoptosis (*P* < 0.001, Fig. 4C–E), which were similar to PSMC2. In contrast, cell migration, which to some extent may be responsible for tumor metastasis, was also evaluated. As illustrated in Fig. 4F, G, knockdown of GNG4 significantly restrained cell migration ability of GBC-SD cells, as indicated by both wound-healing and Transwell assays (*P* < 0.01 for wound-healing assay, *P* < 0.001 for Transwell assay). More significantly, it was subsequently shown that further knockdown of PSMC2 in GNG4 knockdown cells could affect cellular functions including cell proliferation, colony formation, cell apoptosis and migration (*P* < 0.001, Fig. 4C–G), indicative of the role of PSMC2/GNG4 axis in the development of GBC. Furthermore, as shown in Fig. S5, similar results were also obtained in SGC-996 cells.

### PSMC2 suppressed tumor growth in vivo and mechanism screening

**Fig. 5:**
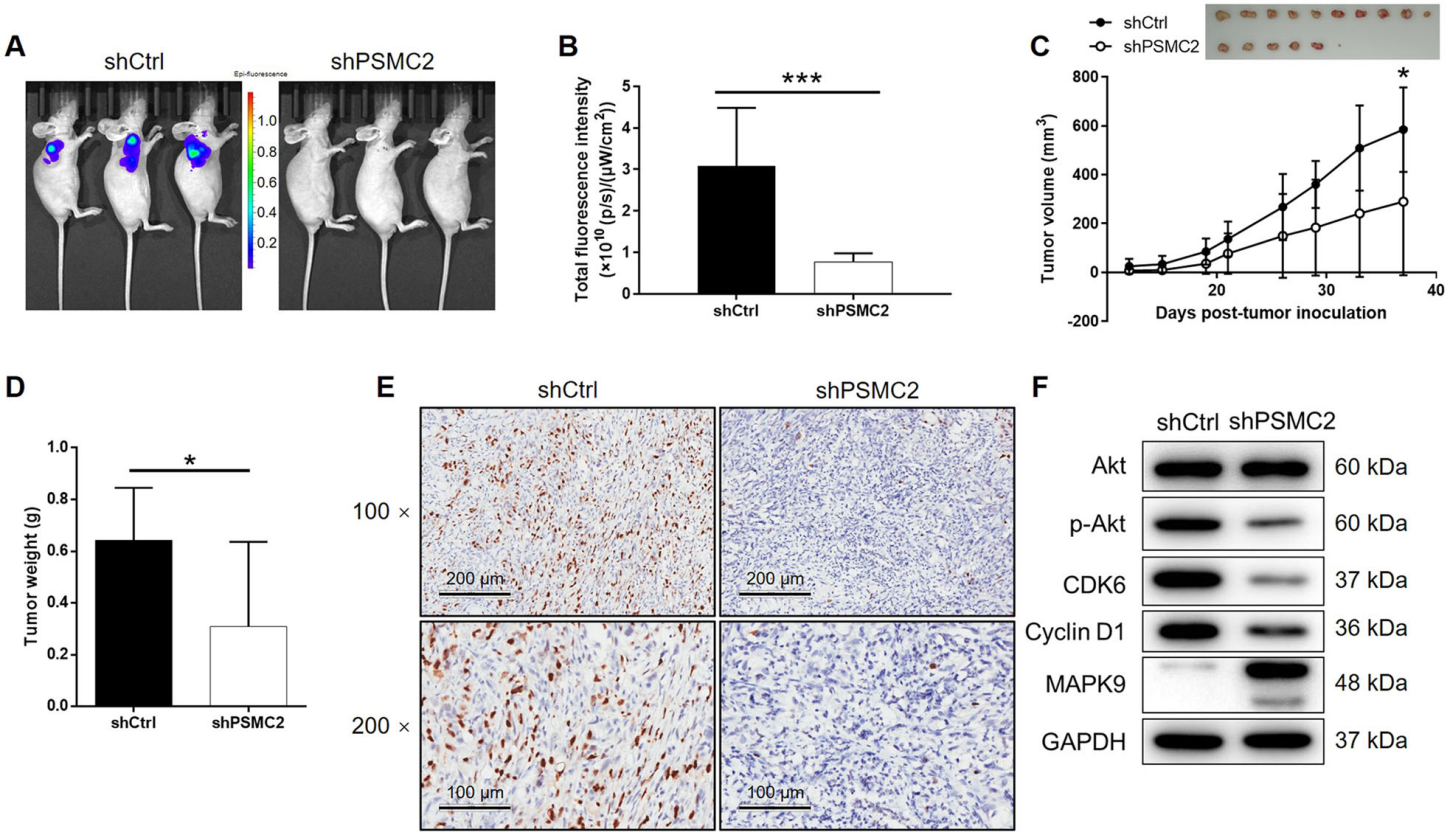
PSMC2 knockdown inhibited GBC development in vivo. **A** In vivo imaging was performed to evaluate the tumor burden in mice of shPSMC2 and shCtrl groups at day 37 post-tumor-inoculation. **B** The fluorescence intensity was scanned and used as a representation of tumor burden in mice of shPSMC2 and shCtrl groups. **C** 12 days post-injection of GBC-SD cells with or without PSMC2 knockdown, the volume of tumors formed in mice was measured and calculated at indicated time intervals. **D** Mice were sacrificed at day 37 post-injection, and the tumors were removed for collecting photos (inset of **C**) and weighing. **E** After removing the tumors, the Ki67 index was evaluated by IHC staining as a representative of tumor growth activity. **F** The expression of Akt, p-Akt, CDK6, Cyclin D1, and MAPK9 was detected by western blotting in GBC-SD cells with or without PSMC2 knockdown. Data were shown as mean ± SD. *P < 0.05, ***P < 0.001.

Following the successful construction of a mouse-based model by injection of GBC-SD cells with or without PSMC2 knockdown, the results of in vivo fluorescnece imaging showed markedly weaker total fluorescence intensity, as well as smaller tumor burden, in shPSMC2 group (*P* < 0.001, Fig. 5A, B). Meanwhile, the reduced number and lighter weight of solid tumors in the shPSMC2 group also suggested that tumor growth decreased upon the silencing of PSMC2 (*P* < 0.001, Fig. 5C, D). The lower Ki67 index detected in the tumors removed from mice of shPSMC2 groups supported the above observations (Fig. 5E). Similarly, in vivo study was also carried out using cell models with GNG4 knockdown (Fig. S6) or simultaneous PSMC2 knockdown and GNG4 knockdown (Fig. S7), all showing similar results and emphasizing the role of PSMC2/GNG4 axis in GBC.

Moreover, the levels of several familiar key molecules in cancer-associated signaling pathways were detected using western blotting as a means to further illustrate the regulatory mechanism of PSMC2 on GBC. As indicated by Fig. 5F, the phosphorylation level of Akt and the expression levels of CDK6 and CCNI1 were appreciably downregulated by PSMC2 knockdown, and MAPK9 was upregulated, which indicated their potential connection to PSMC2-induced regulation of GBC.

## Discussion

As the most frequent form of ubiquitin-mediated proteasome, 26 S proteasome consists of a 20 S core granule and two 19 S regulatory granules^20,21^. In addition, 19 S subunit regulates the whole proteasome through the unique components of its corresponding 19 proteins. As an important component of the 19 S subunit, PSMC2 has a prominent function of binding to ATP, nucleotide, nucleoside triphosphates, and hydrolase. Furthermore, PSMC2 is a key participant in the degradation of intracellular proteins. In 2012, PSMC2 was identified as one of the potential candidate genes for human cancers^15^. Song et al. showed that the expression of PSMC2 was upregulated in osteosarcoma, and knockdown of PSMC2 had the ability to inhibit proliferation and movement of osteosarcoma cells^18^. Moreover, Li et al. demonstrated that PSMC2 was not only a target of miR-630 to promote cell proliferation, migration, and invasion in osteosarcoma, but also was associated with poor prognosis^19^. Nevertheless, the relationship between PSMC2 and GBC has not been reported and still remains unknown.

The clinical significance, biological behavior, and potential molecular mechanisms of PSMC2 in GBC were described in this study. First, the association between PSMC2 expression and tumor grade of GBC was determined, which has important clinical significance. Kaplan–Meier survival analysis confirmed that high PSMC2 expression was significantly associated with poor prognosis in GBC patients. The detection of cell phenotypes of GBC cells with or without PSMC2 knockdown showed that GBC cells with low PSMC2 expression had slower proliferation rate, weaker colony formation ability, and were more prone to apoptosis. Consistently, knockdown of PSMC2 in GBC cells caused weaker tumorigenicity and the tumors grew more slowly in vivo. Moreover, knockdown of PSMC2 resulted in upregulation of apoptosis-related proteins CD40L, FasL, IGFBP-5, IGFBP-6, and P27, as well as downregulation of STNF-R2, TNF-A, and XIAP, which may be the mechanism of PSMC2 regulating apoptosis. These results suggested that PSMC2 has oncogene-like characteristics in GBC. The potential downstream of PSMC2 was screened through RNA-seq in this study. As a member in one of the most DEGs enriched signaling pathways, GNG4 was predicted to have an interaction with PSMC2 and recognized as a potential key factor in the PSMC2 induced regulation of GBC.

G protein is a type of protein that could couple to receptors on cell membranes and transducing signals such as neurotransmitters and hormones to effectors^22,23^. Generally, the structure of G protein consists of 3 subunits (α, β, and γ subunit), which were encoded by corresponding genes^22,24^. As one of the γ subunits of G protein, GNG4 (G protein subunit gamma 4) has been reported as a regulator and transducer of a variety of transmembrane signaling systems^25^. Currently, the research concerning the biological functions of GNG4 is still very limited and mainly focused on neurodegenerative diseases such as Alzheimer’s disease. The study of Yokoyama et al. delineated the decrease of GNG4 expression along with advancing age, and revealed its potential role in cognitive decline in normal aging^26^. As for the relationship between GNG4 and human cancers, Pal et al. identified GNG4 as a potential tumor-suppressor in glioblastoma, which was seriously methylated and downregulated. As indicated, the mechanism underlying the GNG4-silencing driven promotion of glioblastoma may be the inhibition of SDF1α/CXCR4 signaling^27^.

To the best of our knowledge, the association between GNG4 and GBC is still largely unknown. Overexpression of GNG4 in GBC tissues and cell lines was observed in this study, which was further significantly associated with more advanced tumor grade and poorer prognosis. Knockdown of GNG4 inhibited GBC cell proliferation, colony formation, and cell migration while promoting cell apoptosis. More importantly, knockdown of PSMC2 could lead to the downregulation of GNG4, and vice versa, indicating the interaction between PSMC2 and GNG4. Results of co-IP provided more direct evidence of the interaction between PSMC2 and GNG4. Furthermore, simultaneous knockdown of PSMC2 and GNG4 exhibited stronger inhibition of cell proliferation, colony formation and cell migration and promotion of cell apoptosis than mere GNG4 knockdown.

The biological behavior of Akt-mediated signal transduction in regulating the proliferation, differentiation, apoptosis, and migration of tumor cells has been widely explored^28–30^. For example, Goel et al. put forward that the activation of Akt signaling pathway is associated with the development and metastasis of GBC^31^. In this study, we found that knockdown of PSMC2 led to a significant downregulation of phosphorylated Akt, indicating that PSMC2 activated the Akt signal in GBC. In addition, CDK6 and Cyclin D1 are known to be essential regulators of the cycle and proliferation^32–34^, whose expression is of great significance to the prognosis of GBC^35,36^. Consistently, our results showed significant downregulation of CDK6 and Cyclin D1 upon knockdown of PSMC2. MAPK9, also known as JNK2, is one of three isoenzymes of c-Jun N-terminal kinase (JNK), which has been implicated in the regulation of human cancer^37^. For example, Pang et al. reported that JNK2 acted as the target to mediate the miR-146a induced increase of cisplatin resistance in non-small cell lung cancer^38^. In addition, through JNK signaling, Shikonin can induce apoptosis and arrest of cycle G0/G1 phase of GBC cells^39^. The present study demonstrated that the knockdown of PSMC2 suppressed the expression of JNK2, indicating that JNK2 is a potential downstream target. Despite of these results, the underlying mechanism of the regulatory effects of PSMC2 on the development and metastasis of GBC is still not clear, which will be the focus of future work.

In conclusion, PSMC2 possesses the ability to promote the development of GBC by targeting GNG4. Therefore, PSMC2 may exert a promoting role in GBC, which may act a potential prognostic index and therapeutic target for the treatment of GBC.

## Supplementary information

Agreement from all authors

Table S1

Table S2

Table S3

Table S4

Table S5

Table S6

Supplementary figure legends

Figure S1

Figure S2

Figure S3

Figure S4

Figure S5

Figure S6

Figure S7
