# Supplementary material for: High expression of PSMC2 promotes gallbladder cancer through regulation of GNG4 and predicts poor prognosis
Source: Oncogenesis. 2021 May 20;10(5):43. doi: 10.1038/s41389-021-00330-1 (PMC8138011; doi:10.1038/s41389-021-00330-1)
Supplement: Supplementary file 1 — Agreement from all authors [file 41389_2021_330_MOESM1_ESM.pdf]

Outlook 搜索 现在开会

使用 Microsoft 的新浏览器提高速度、安全并受到隐私保护。 立即下载

新建邮件 答复 删除 存档 垃圾邮件 整理 移至 分类 推迟 ···

文件夹
收件箱 700
垃圾邮件 4
草稿 2
已发送邮件
已删除邮件
存档
便笺 2
Notes
对话历史记录
新建文件夹

Re: add authors of this article

将消息翻译为 简体中文 | 始终不翻译 英语

白猫~ <394579360@qq.com>
周三 2021/3/31 15:03
收件人: 你

I agree with the decision to add Xing Gu as the co-first authors of this article.

---Original---

From: "精灵 精灵"<littlespirit1980@hotmail.com>
Date: Wed, Mar 31, 2021 13:59 PM
To: "394579360@qq.com"<394579360@qq.com>;
Subject: add authors of this article

Our manuscript submitted to Oncogenesis 2020.4.29 (ONCSIS-20-0288), and reviewers' comments returned on 2020.5.9. As a lot of experiments and modifications were done by Xing Gu during the revision, we decided to add Xing Gu as the co-first authors of this article. The decision is agreed by all authors of this work.

答复 | 转发

Re: add authors of this article

将消息翻译为 简体中文 | 始终不翻译 英语

幽灵剑客 <410098547@qq.com>
周三 2021/3/31 14:14
收件人: 你

I agree with the decision to add Xing Gu as the co-first authors of this article.

---Original---

From: "精灵 精灵"<littlespirit1980@hotmail.com>
Date: Wed, Mar 31, 2021 14:07 PM
To: "410098547@qq.com"<410098547@qq.com>;"284409937@qq.com"<284409937@qq.com>;"yudandan@hust.edu.cn" <yudandan@hust.edu.cn>;
Subject: add authors of this article

Our manuscript submitted to Oncogenesis 2020.4.29 (ONCSIS-20-0288), and reviewers' comments returned on 2020.5.9. As a lot of experiments and modifications were done by Xing Gu during the revision, we decided to add Xing Gu as the co-first authors of this article. The decision is agreed by all authors of this work.

答复 | 转发

Re: add authors of this article

将消息翻译为 简体中文 | 始终不翻译 英语

toji <284409937@qq.com>
周三 2021/3/31 14:11
收件人: 你

I agree with the decision to add Xing Gu as the co-first authors of this article.

---Original---

From: "精灵 精灵"<littlespirit1980@hotmail.com>
Date: Wed, Mar 31, 2021 14:07 PM
To: "410098547@qq.com"<410098547@qq.com>;"284409937@qq.com"<284409937@qq.com>;"yudandan@hust.edu.cn" <yudandan@hust.edu.cn>;
Subject: add authors of this article

Our manuscript submitted to Oncogenesis 2020.4.29 (ONCSIS-20-0288), and reviewers' comments returned on 2020.5.9. As a lot of experiments and modifications were done by Xing Gu during the revision, we decided to add Xing Gu as the co-first authors of this article. The decision is agreed by all authors of this work.

答复 | 转发

Re:add authors of this article

将消息翻译为 简体中文 | 始终不翻译 英语

于丹丹 <yudandan@hust.edu.cn>
周三 2021/3/31 23:07
收件人: 你

I agree with the decision to add Xing Gu as the co-first authors of this article.

发件人: "精灵 精灵" <littlespirit1980@hotmail.com>
发送日期: 2021-03-31 14:07:14
收件人: "410098547@qq.com" <410098547@qq.com>, "284409937@qq.com" <284409937@qq.com>, "yudandan@hust.edu.cn" <yudandan@hust.edu.cn>
抄送人:
主 题: add authors of this article

Our manuscript submitted to Oncogenesis 2020.4.29 (ONCSIS-20-0288), and reviewers' comments returned on 2020.5.9. As a lot of experiments and modifications were done by Xing Gu during the revision, we decided to add Xing Gu as the co-first authors of this article. The decision is agreed by all authors of this work.

答复 | **转发**
