# Supplementary material for: High expression of PSMC2 promotes gallbladder cancer through regulation of GNG4 and predicts poor prognosis
Source: Oncogenesis. 2021 May 20;10(5):43. doi: 10.1038/s41389-021-00330-1 (PMC8138011; doi:10.1038/s41389-021-00330-1)
Supplement: Supplementary file 2 — Table S1 [file 41389_2021_330_MOESM2_ESM.docx]

Table S1 Antibodies used in western blotting and IHC

| Primary antibodies | Dilution in WB | Source species | Company | Catalog No. |
| --- | --- | --- | --- | --- |
| PSMC2 | 1:1000 | Mouse | Santa Cruz | SC-166972 |
| GAPDH | 1:3000 | Rabbit | Bioworld | AP0063 |
| c-Myc(MYC) | 1:1000 | Rabbit | CST | 5605 |
| MAPK9 | 1:1500 | Rabbit | abcam | ab76125 |
| RHOU | 1:1000 | Rabbit | Abcam | ab80315 |
| THBS1 | 1:1000 | Rabbit | CST | 37879 |
| DYKDDDDK Tag* | 1:50/1:1000 | Rabbit | CST | 14793 |
| HA | 1:25/1:3000 | Rabbit | abcam | ab9110 |
| *: DYKDDDDK Tag binds to same epitope as Sigma's Anti-FLAG® M2 Antibody | | | | |
| Primary antibodies | Dilution in IHC | Source species | Company | Catalog No. |
| GNG4 | 1:100 | Rabbit | abcam | ab238868 |
| PSMC2 | 1:100 | Rabbit | Santa Cruz | SC-166972 |
| Ki67 | 1:200 | Rabbit | abcam | ab16667 |
|  |  |  |  |  |
|  |  |  |  |  |
| Secondary antibody | Dilution |  | Company | Catalog No. |
| HRP Goat Anti-Rabbit IgG (WB) | 1:3000 |  | Beyotime | A0208 |
| HRP Goat Anti-Mouse IgG (WB) | 1:3000 |  | Beyotime | A0216 |
| HRP Goat Anti-Rabbit IgG (IHC) | 1:200 |  | Abcam | Ab111909 |
