# Supplementary material for: High expression of PSMC2 promotes gallbladder cancer through regulation of GNG4 and predicts poor prognosis
Source: Oncogenesis. 2021 May 20;10(5):43. doi: 10.1038/s41389-021-00330-1 (PMC8138011; doi:10.1038/s41389-021-00330-1)
Supplement: Supplementary file 3 — Table S2 [file 41389_2021_330_MOESM3_ESM.docx]

Table S2 The target sequences and shRNA sequences

| Gene | No. | Target sequence (5'-3') | shRNA sequences (5'-3') |
| --- | --- | --- | --- |
| PSMC2 | Human-PSMC2-1-a | GCCAGGGAGATTGGATAGAAA | ccggGCCAGGGAGATTGGATAGAAAttcaagagaTTTCTATCCAATCTCCCTGGCtttttg |
|  | Human-PSMC2-1-b | GCCAGGGAGATTGGATAGAAA | aattcaaaaaGCCAGGGAGATTGGATAGAAAttcaagagaTTTCTATCCAATCTCCCTGGC |
| GNG4 | Pbr22352-a | AGCCAGGAAAGCTGTGGAGCA | CCGGAGCCAGGAAAGCTGTGGAGCACTCGAGTGCTCCACAGCTTTCCTGGCTTTTTTG |
|  | Pbr22352-b | AGCCAGGAAAGCTGTGGAGCA | AATTCAAAAAAGCCAGGAAAGCTGTGGAGCACTCGAGTGCTCCACAGCTTTCCTGGCT |
| GNG4 | Pbr22353-a | TGAAAGAGGGCATGTCTAATA | CCGGTGAAAGAGGGCATGTCTAATACTCGAGTATTAGACATGCCCTCTTTCATTTTTG |
|  | Pbr22353-b | TGAAAGAGGGCATGTCTAATA | AATTCAAAAATGAAAGAGGGCATGTCTAATACTCGAGTATTAGACATGCCCTCTTTCA |
| GNG4 | Pbr22354-a | GCGGGAAGATCCTCTCATCAT | CCGGGCGGGAAGATCCTCTCATCATCTCGAGATGATGAGAGGATCTTCCCGCTTTTTG |
|  | Pbr22354-b | GCGGGAAGATCCTCTCATCAT | AATTCAAAAAGCGGGAAGATCCTCTCATCATCTCGAGATGATGAGAGGATCTTCCCGC |
|  |  |  |  |
|  |  |  |  |
|  |  |  |  |
|  |  |  |  |
|  |  |  |  |
|  |  |  |  |
|  |  |  |  |
|  |  |  |  |
|  |  |  |  |
|  |  |  |  |
|  |  |  |  |
|  |  |  |  |
|  |  |  |  |
|  |  |  |  |
|  |  |  |  |
|  |  |  |  |
|  |  |  |  |
|  |  |  |  |
|  |  |  |  |
|  |  |  |  |
|  |  |  |  |
|  |  |  |  |
|  |  |  |  |
|  |  |  |  |
|  |  |  |  |
|  |  |  |  |
|  |  |  |  |
|  |  |  |  |
|  |  |  |  |
|  |  |  |  |
|  |  |  |  |
|  |  |  |  |
|  |  |  |  |
|  |  |  |  |
|  |  |  |  |
|  |  |  |  |
|  |  |  |  |
|  |  |  |  |
|  |  |  |  |
|  |  |  |  |
|  |  |  |  |
|  |  |  |  |
|  |  |  |  |
|  |  |  |  |
|  |  |  |  |
|  |  |  |  |
|  |  |  |  |
|  |  |  |  |
|  |  |  |  |
|  |  |  |  |
|  |  |  |  |
|  |  |  |  |
