# Supplementary material for: High expression of PSMC2 promotes gallbladder cancer through regulation of GNG4 and predicts poor prognosis
Source: Oncogenesis. 2021 May 20;10(5):43. doi: 10.1038/s41389-021-00330-1 (PMC8138011; doi:10.1038/s41389-021-00330-1)
Supplement: Supplementary file 4 — Table S3 [file 41389_2021_330_MOESM4_ESM.docx]

Table S3 Primers used in qPCR

| Gene | Forward primer sequence (5’-3’) | Reverse primer sequence (5’-3’) |
| --- | --- | --- |
| GAPDH | TGACTTCAACAGCGACACCCA | CACCCTGTTGCTGTAGCCAAA |
| PSMC2 | CAGCACTCTGGGATTTGGCT | TTTCTATCCACGCCCACTCTC |
| MAPK9 | CTCTGCGTCACCCATACATCA | TCTTTCTTCCAACTGGGCATC |
| LMNB1 | CCCAGTTGGAAGCCTCCTTA | GCGAAACTCCAAGTCCTCAG |
| BMP2 | TATCGCAGGCACTCAGGTCA | CCACTCGTTTCTGGTAGTTCTTC |
| RHOU | CGCCTCCTACATCGAGTGTT | GACTTCTTTGGCTGTTGCTGA |
| CUL3 | GCCTTGACAAATCAACGGAA | ACATGCAACCAAGGTCTTCTG |
| CDC42 | GACAGATTACGACCGCTGAGTT | GGAGTCTTTGGACAGTGGTGAG |
| ZNF33B | CAACCTTGTCTCAGTGGGGTAT | AAAGCTCTGGCTTGGGAATT |
| MYC | TGCTGCCAAGAGGGTCAAGT | GCTCCGTTTTAGCTCGTTCC |
| ID2 | CCGTGAGGTCCGTTAGGAAA | TGAGCTTGGAGTAGCAGTCG |
| CBX5 | CTTCAGAGGATGAGGAGGAGTATG | GCTCAGGGCAATCCAAGTTTT |
| RHOV | CAAGAGCAGCCTCATCGTCA | TCCACCAGGACTTGCACAGA |
| FOS | CAGACTACGAGGCGTCATCC | TCTGCGGGTGAGTGGTAGTA |
| RPS6KA3 | ATTGGCACGAATAGGTAGCG | GCATCTTTGACACCAGGTCC |
| THBS1 | TGTTCTCTACTGGCTTTATGTCA | GGCCTGAGCAACTCAGTCTT |
| NFKBIA | CTCCATCCTGAAGGCTACCAA | GCACCCAAGGACACCAAAAG |
| YAP1 | TGGCAAAGACATCTTCTGGTC | CATATTCTGCTGCACTGGTGG |
| SNAI2 | AAAGCCAAACTACAGCGAACTG | TGGTATGACAGGCATGGAGTAA |
| PTEN | AAGACCATAACCCACCACAGC | ACACCAGTTCGTCCCTTTCC |
| MMP7 | GACTTCCAAAGTGGTCACCTACA | CAGTTCCCCATACAACTTTCCT |
| TMPO | CCCCTATGAAGCATCTACACCA | GCCAAGGGAACATACTTAGGAAC |
