# Supplementary material for: High expression of PSMC2 promotes gallbladder cancer through regulation of GNG4 and predicts poor prognosis
Source: Oncogenesis. 2021 May 20;10(5):43. doi: 10.1038/s41389-021-00330-1 (PMC8138011; doi:10.1038/s41389-021-00330-1)
Supplement: Supplementary file 5 — Table S4 [file 41389_2021_330_MOESM5_ESM.docx]

Table S4 Relationship between PSMC2 expression and tumor characteristics in patients with gallbladder cancer analyzed by Spearman rank correlation analysis

| Tumor characteristics | index |  |
| --- | --- | --- |
| Grade | Spearman correlation | 0.606 |
|  | Significance (two tailed) | <0.001 |
|  | n | 78 |
