# Supplementary material for: High expression of PSMC2 promotes gallbladder cancer through regulation of GNG4 and predicts poor prognosis
Source: Oncogenesis. 2021 May 20;10(5):43. doi: 10.1038/s41389-021-00330-1 (PMC8138011; doi:10.1038/s41389-021-00330-1)
Supplement: Supplementary file 6 — Table S5 [file 41389_2021_330_MOESM6_ESM.docx]

Table S5 Expression patterns of GNG4 in gallbladder cancer tissues and normal tissues revealed in immunohistochemistry analysis

| GNG4 expression | Tumor tissue | | Normal tissue | |
| --- | --- | --- | --- | --- |
|  | Cases | Percentage | Cases | Percentage |
| Low | 36 | 46.2% | 14 | 87.5% |
| High | 42 | 53.8% | 2 | 12.5% |

*P* < 0.001
