# Supplementary material for: High expression of PSMC2 promotes gallbladder cancer through regulation of GNG4 and predicts poor prognosis
Source: Oncogenesis. 2021 May 20;10(5):43. doi: 10.1038/s41389-021-00330-1 (PMC8138011; doi:10.1038/s41389-021-00330-1)
Supplement: Supplementary file 7 — Table S6 [file 41389_2021_330_MOESM7_ESM.docx]

Table S6 Relationship between GNG4 expression and tumor characteristics in patients with gallbladder cancer

| Features | No. of patients | GNG4 expression | | *P* value |
| --- | --- | --- | --- | --- |
|  |  | low | high |  |
| All patients | 78 | 36 | 42 |  |
| Age (years) |  |  |  | 0.097 |
| <65 | 35 | 20(55.6%) | 15(35.7%) |  |
| ≥65 | 42 | 16(44.4%) | 26(61.9%) |  |
| Gender |  |  |  | 0.599 |
| Male | 24 | 10(27.8%) | 14(33.3%) |  |
| Female | 54 | 26(72.2%) | 28(66.7%) |  |
| Grade |  |  |  | 0.040 |
| I | 4 | 3(8.3%) | 1(2.4%) |  |
| II | 30 | 17(47.2%) | 13(31.0%) |  |
| III | 44 | 16(44.5%) | 28(66.6%) |  |
| T [Infiltrate](D:/360%E5%AE%89%E5%85%A8%E6%B5%8F%E8%A7%88%E5%99%A8%E4%B8%8B%E8%BD%BD/Dict/8.4.0.0/resultui/html/index.html#/javascript:;) |  |  |  | 0.818 |
| T1 | 6 | 4(11.1%) | 2(4.8%) |  |
| T2 | 22 | 8(22.2%) | 14(33.3%) |  |
| T3 | 30 | 13(36.1%) | 17(40.5%) |  |
| T4 | 2 | 1(2.8%) | 1(2.4%) |  |
| [lymphatic](D:/360%E5%AE%89%E5%85%A8%E6%B5%8F%E8%A7%88%E5%99%A8%E4%B8%8B%E8%BD%BD/Dict/8.4.0.0/resultui/html/index.html#/javascript:;) [metastasis](D:/360%E5%AE%89%E5%85%A8%E6%B5%8F%E8%A7%88%E5%99%A8%E4%B8%8B%E8%BD%BD/Dict/8.4.0.0/resultui/html/index.html#/javascript:;) (N) |  |  |  | 0.127 |
| N0 | 45 | 19(52.8%) | 26(61.9%) |  |
| N1 | 7 | 6(16.7%) | 1(2.4%) |  |
| N2 | 6 | 3(8.3%) | 3(7.1%) |  |
| AJCC stage |  |  |  | 0.960 |
| 1 | 5 | 3(8.3%) | 2(4.8%) |  |
| 2 | 12 | 3(8.3%) | 9(21.4%) |  |
| 3 | 18 | 10(27.8%) | 8(19.0%) |  |
| 4 | 16 | 6(16.7%) | 10(23.8%) |  |
| Tumor size |  |  |  | 0.297 |
| <4.3cm | 38 | 20(55.6%) | 18(42.9%) |  |
| ≥4.3cm | 37 | 15(41.7%) | 22(52.4%) |  |
| Metastasis |  |  |  | 0.077 |
| M0 | 68 | 34(94.4%) | 34(81.0%) |  |
| M1 | 10 | 2(5.6%) | 8(19.0%) |  |
| Lymph node positive |  |  |  | 0.089 |
| =0 | 45 | 19(52.8%) | 26(61.9%) |  |
| >0 | 13 | 9(25%) | 4(9.5%) |  |
