# Supplementary material for: High expression of PSMC2 promotes gallbladder cancer through regulation of GNG4 and predicts poor prognosis
Source: Oncogenesis. 2021 May 20;10(5):43. doi: 10.1038/s41389-021-00330-1 (PMC8138011; doi:10.1038/s41389-021-00330-1)
Supplement: Supplementary file 8 — Supplementary figure legends [file 41389_2021_330_MOESM8_ESM.docx]

**Figure S1.** The transfection efficiencies of shPSMC2 and shCtrl in GBC-SD and SGC-996 cells were evaluated through observing the fluorescence of GFP on lentivirus vector.

**Figure S2.** Human Apoptosis Antibody Array was utilized to analyze the regulatory ability of PSMC2 on expression of apoptosis-related proteins in GBC-SD cells.

**Figure S3.** (A) The volcano plot of gene expression profiling in SGC-996 cells with or without PSMC2 knockdown. Red dots represented significantly upregulated DEGs. Green dots represented significantly downregulated DEGs. (B) The enrichment of the DEGs in canonical signaling pathways was analyzed by IPA. (C) The enrichment of the DEGs in IPA disease and function was analyzed by IPA. (D) Celigo cell counting assay was performed to evaluate the inhibition of GBC cell proliferation by knockdown of the candidates. Data was shown as mean ± SD. **P* < 0.05

**Figure S4.** (A) The transfection efficiencies of shCtrl, shGNG4, shPSMC2+shGNG4 in GBC-SD cells were evaluated through observing the fluorescence of GFP on lentivirus vector. (B) The knockdown efficiencies of 3 shRNAs designed for GNG4 knockdown were evaluated by qPCR in GBC-SD cells. Data was shown as mean ± SD. ****P* < 0.001

**Figure S5.** Upon transfection of shCtrl, shGNG4 or simultaneous shPSMC2 and shGNG4, SGC-996 cell models were subjected to the detection of cell proliferation by Celigo cell counting assay (A), colony formation (B), cell apoptosis (C), cell migration by wound-healing assay (D) and cell migration by Transwell assay (E). The representative images were selected from at least 3 independent experiments. Data was shown as mean ± SD. **P* < 0.05, ***P* < 0.01, ****P* < 0.001

**Figure S6.** (A) *In vivo* imaging was performed to evaluate the tumor burden in mice of shGNG4 and shCtrl groups at day 37 post tumor-inoculation. (B) The fluorescence intensity was scanned and used as a representation of tumor burden in mice of shGNG4 and shCtrl groups. (C) 12 days post injection of GBC-SD cells with or without GNG4 knockdown, the volume of tumors formed in mice was measured and calculated at indicated time intervals. (D) Mice were sacrificed at day 37 post injection, and the tumors were removed for collecting photos (inset of C) and weighing. Data was shown as mean ± SD. ***P* < 0.01

**Figure S7.** (A) *In vivo* imaging was performed to evaluate the tumor burden in mice of shPSMC2+shGNG4 and shCtrl groups at day 37 post tumor-inoculation. (B) The fluorescence intensity was scanned and used as a representation of tumor burden in mice of shPSMC2+shGNG4 and shCtrl groups. (C) 12 days post injection of GBC-SD cells with or without PSMC2 and GNG4 knockdown, the volume of tumors formed in mice was measured and calculated at indicated time intervals. (D) Mice were sacrificed at day 37 post injection, and the tumors were removed for collecting photos (inset of C) and weighing. Data was shown as mean ± SD. **P* < 0.05, ***P* < 0.01
