# Supplementary figures and images for: High expression of PSMC2 promotes gallbladder cancer through regulation of GNG4 and predicts poor prognosis
Source: Oncogenesis. 2021 May 20;10(5):43. doi: 10.1038/s41389-021-00330-1 (PMC8138011; doi:10.1038/s41389-021-00330-1)

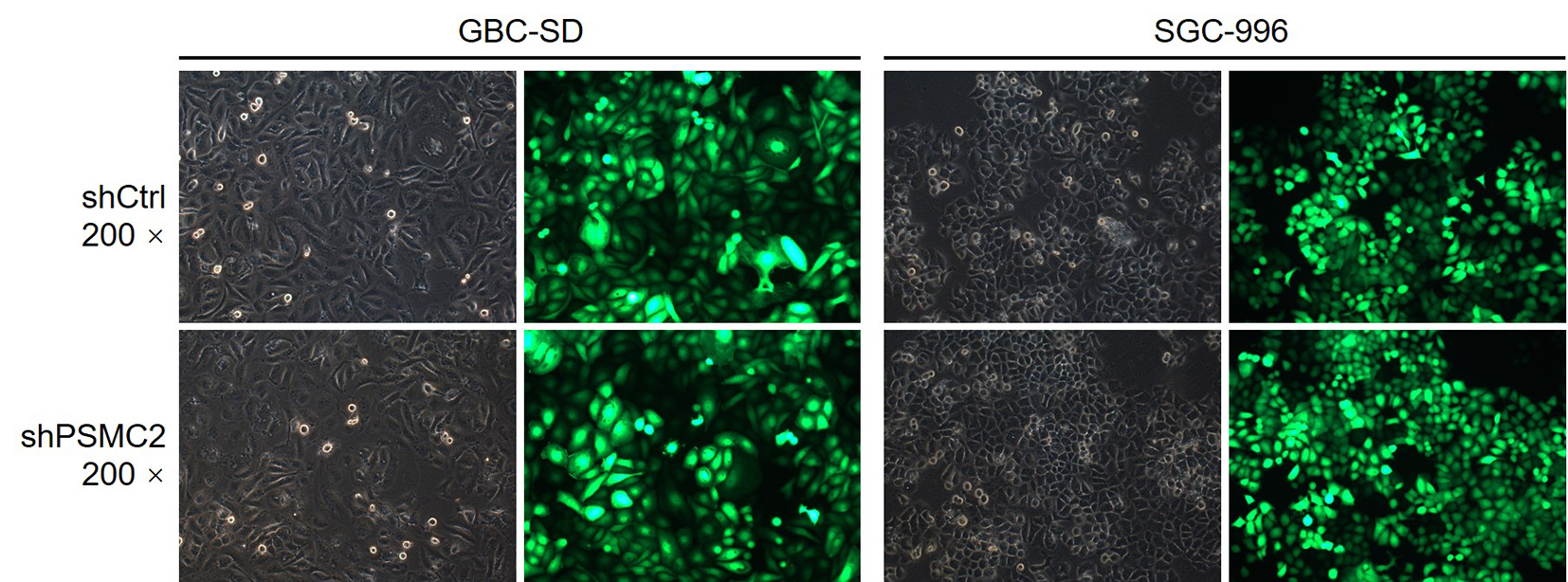

Supplement: Supplementary file 9 — Figure S1 [file 41389_2021_330_MOESM9_ESM.tif]

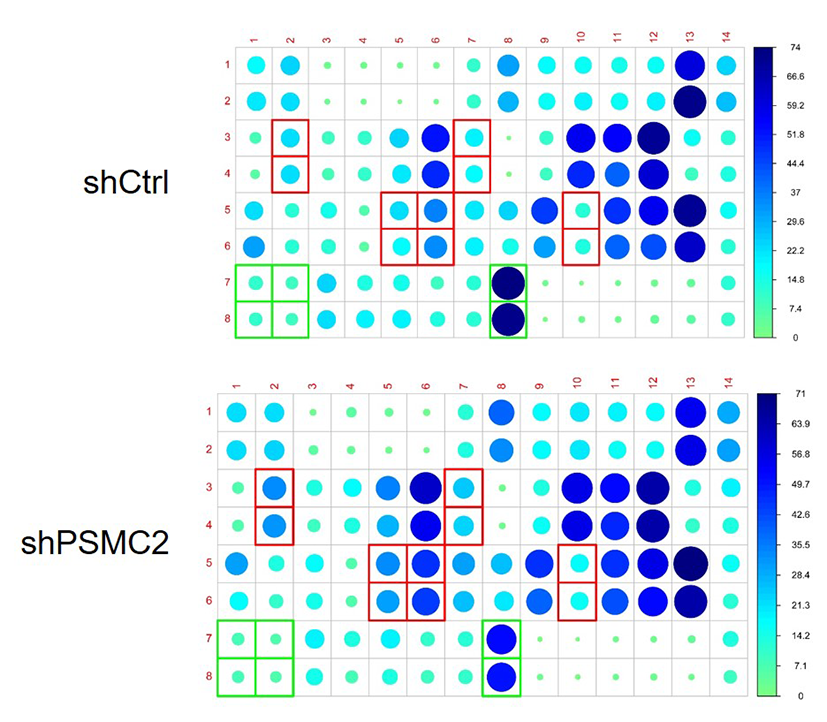

Supplement: Supplementary file 10 — Figure S2 [file 41389_2021_330_MOESM10_ESM.tif]

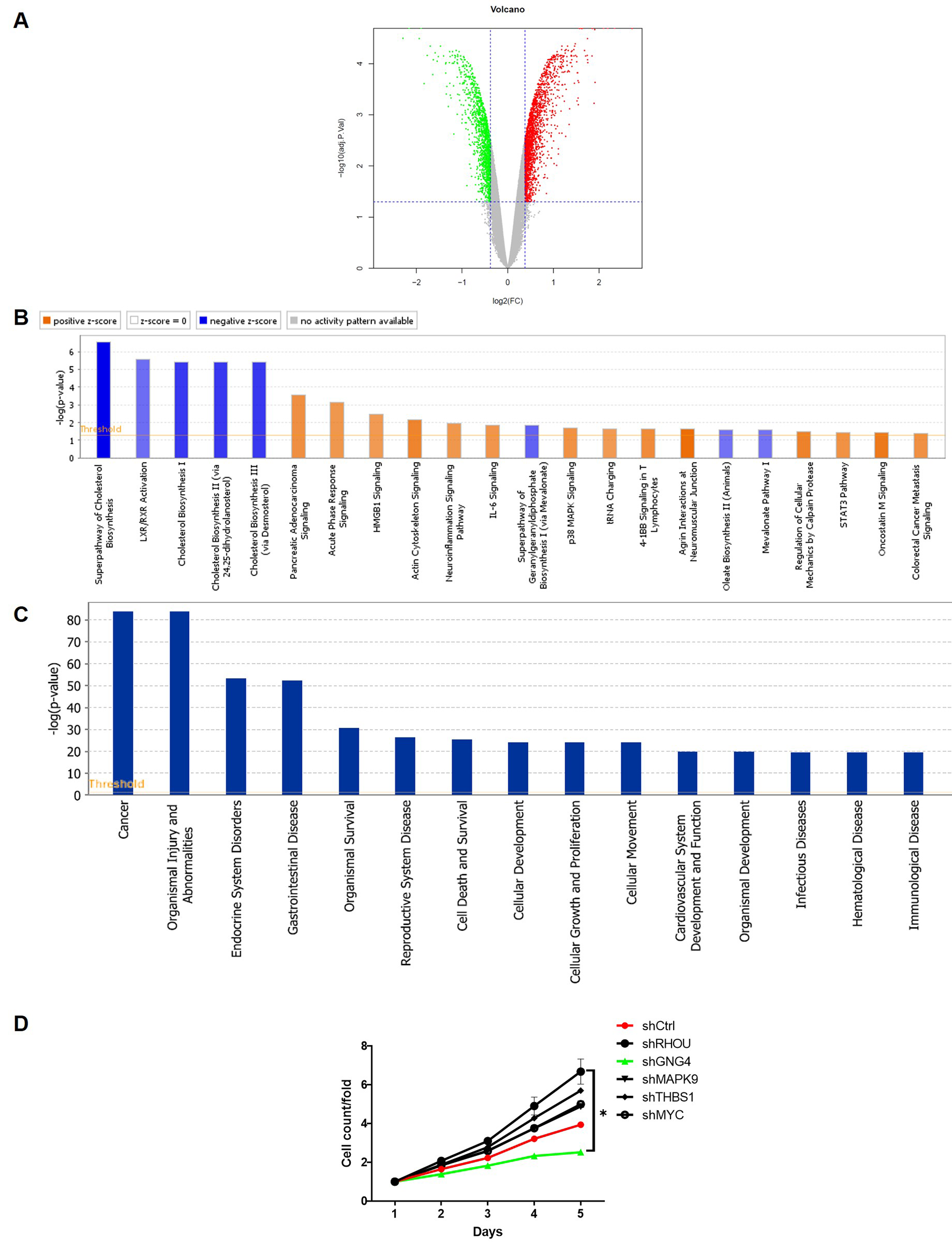

Supplement: Supplementary file 11 — Figure S3 [file 41389_2021_330_MOESM11_ESM.tif]

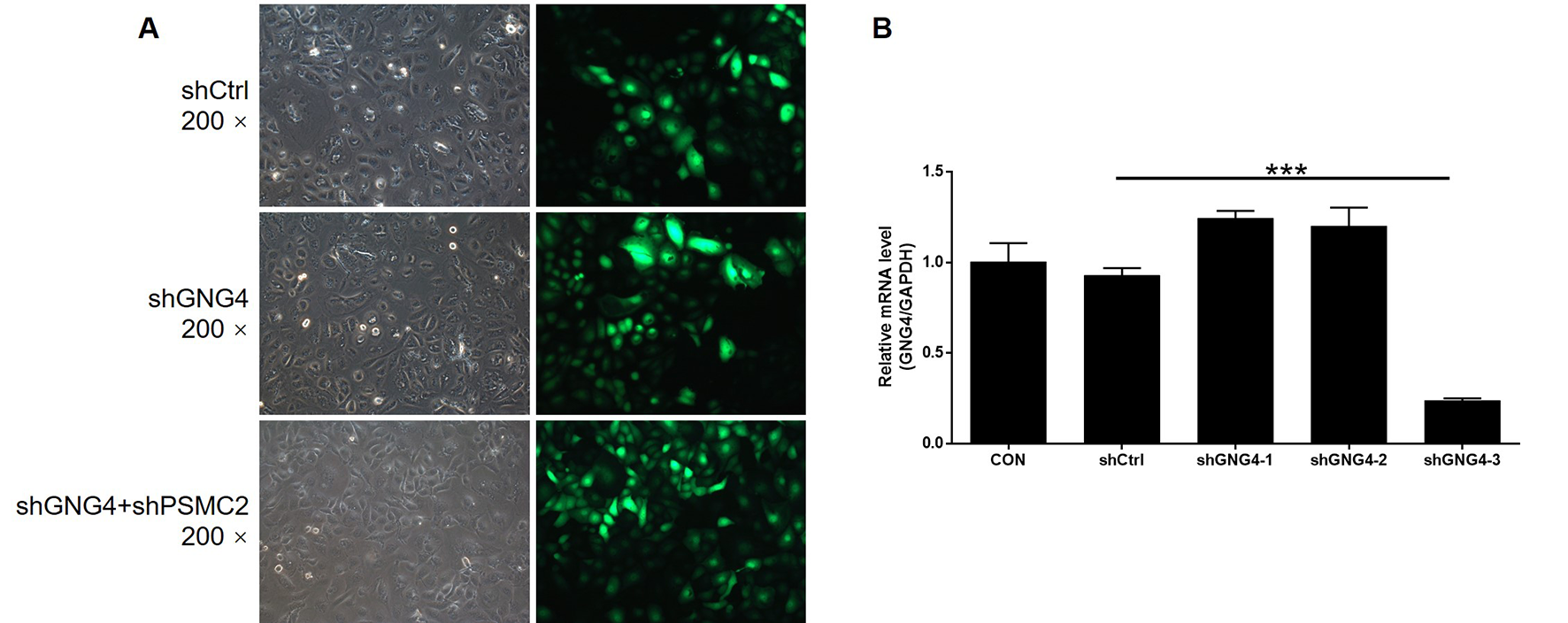

Supplement: Supplementary file 12 — Figure S4 [file 41389_2021_330_MOESM12_ESM.tif]

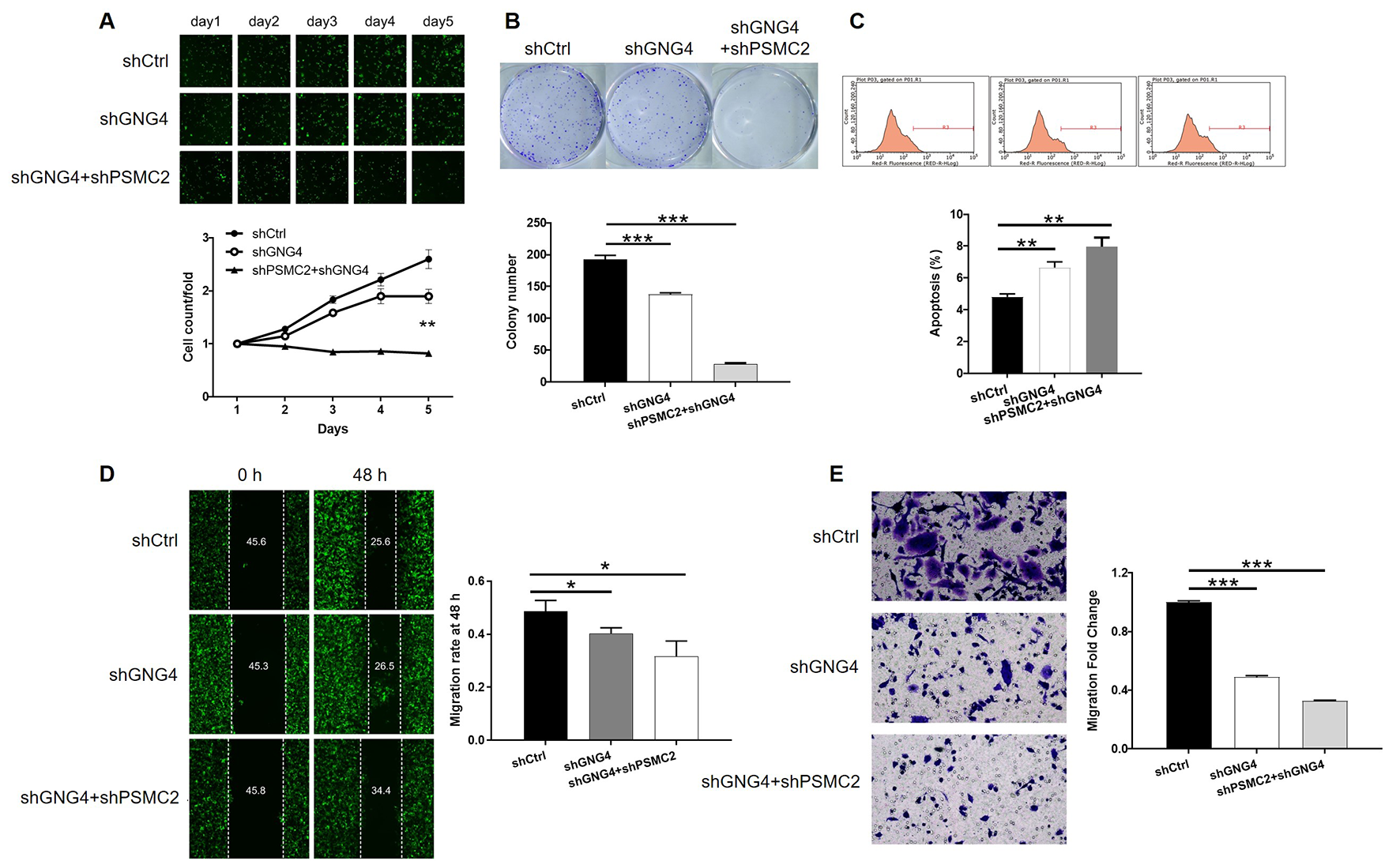

Supplement: Supplementary file 13 — Figure S5 [file 41389_2021_330_MOESM13_ESM.tif]

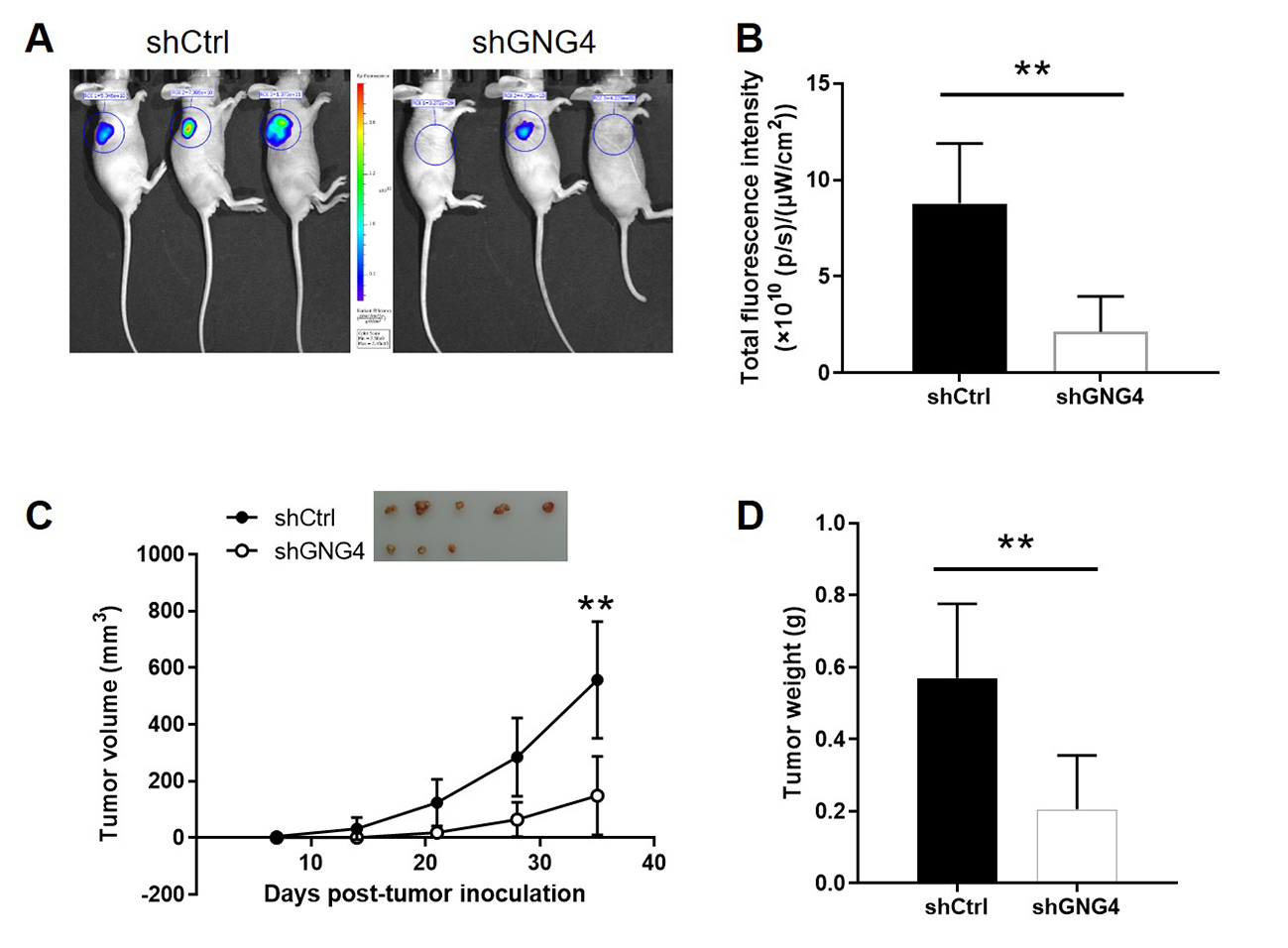

Supplement: Supplementary file 14 — Figure S6 [file 41389_2021_330_MOESM14_ESM.tif]

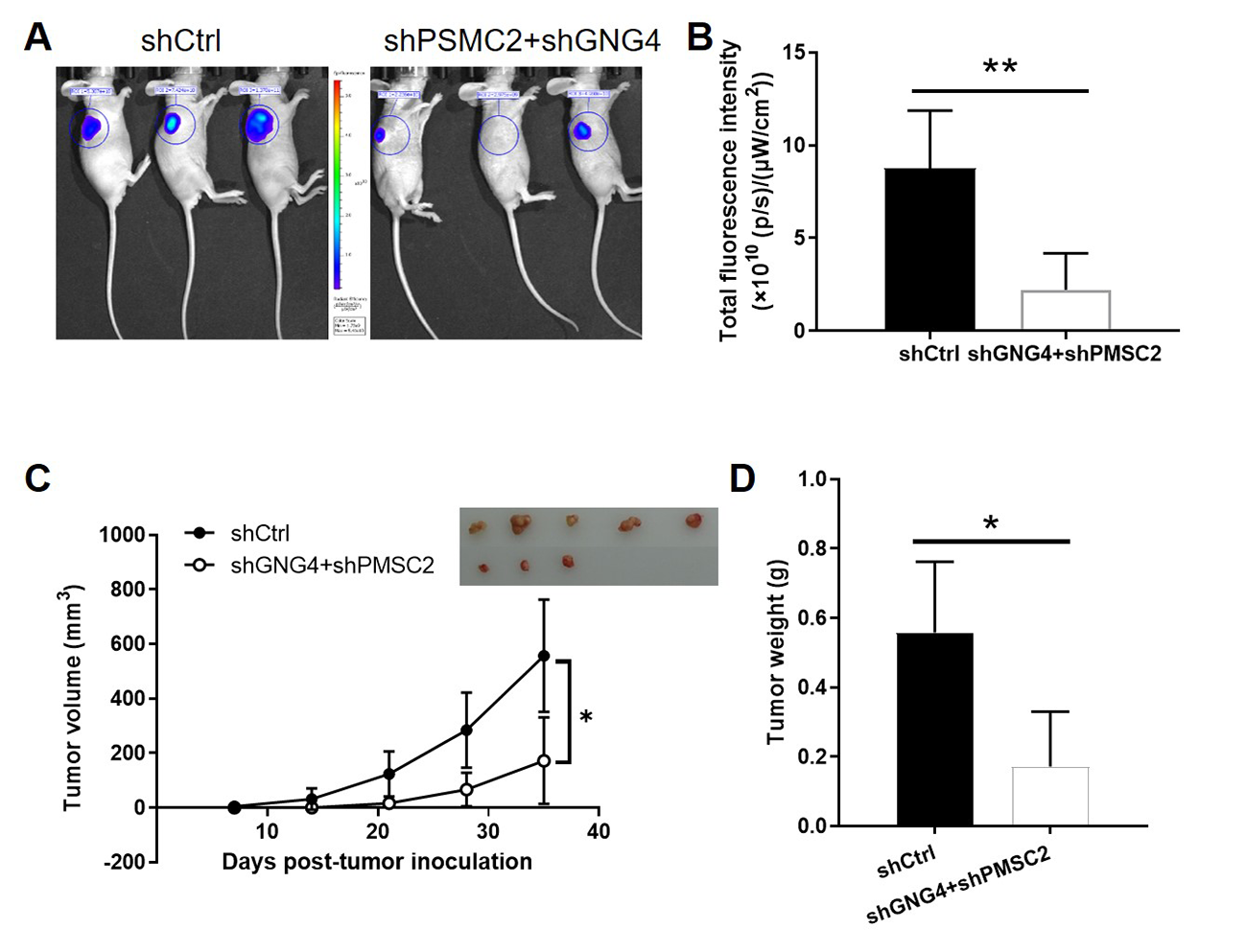

Supplement: Supplementary file 15 — Figure S7 [file 41389_2021_330_MOESM15_ESM.tif]
